# Diphtheria Vaccination Coverage and Correlation With Multidimensional Poverty Among Children in Nigeria

**DOI:** 10.1002/puh2.70214

**Published:** 2026-04-02

**Authors:** Olufemi Olulaja, Rana Jaber

**Affiliations:** ^1^ Department of Health and Kinesiology College of Applied Health Sciences University of Illinois at Urbana‐Champaign Urbana‐Champaign Illinois USA; ^2^ School of Health Studies College of Health and Human Sciences Northern Illinois University DeKalb Illinois USA

**Keywords:** childhood immunization Nigeria, diphtheria vaccine, diphtheria–pertussis–tetanus (DPT) vaccination, multidimensional poverty index

## Abstract

**Introduction:**

Nigeria has witnessed recurrent outbreaks of diphtheria in recent times. This study measured the prevalence of diphtheria–pertussis–tetanus (DPT) vaccination coverage among children aged 12–23 months and its correlation with multidimensional poverty index (MPI) across the 36 Nigeria's states and the federal capital territory.

**Methods:**

State‐level data for DPT vaccination coverage rates and MPI were obtained from the 2021 Multiple Indicator Cluster Survey (MICS)/National Immunization Coverage Survey (NICS) and National Bureau of Statistics, respectively. The correlation between MPI and diphtheria vaccination coverage was examined using SPSS V.28, and alpha level was set to 0.05.

**Results:**

DPT vaccination coverage among children aged 12–23 months is suboptimal at 56%. DPT full vaccination coverage is higher in Southern states than Northern states; all states with ≥80% coverage are in Southern Nigeria, whereas all states with ≤50% coverage are in Northern Nigeria, except for Ogun state (41.3%). Partial DPT vaccination is also higher among Northern states, with Borno state having the highest partial DPT vaccination rate at 27.8%, whereas Ebonyi state had the lowest (0.4%). States with higher MPI had higher proportions of partially vaccinated and unvaccinated children. There is a statistically significant negative correlation between MPI and DPT full vaccination coverage (*r* = −0.534, *p* = <0.001).

**Conclusion:**

DPT vaccination coverage is still low in Nigeria. Northern states had lower vaccination coverage than Southern states. There is a significant negative correlation between DPT vaccination coverage and MPI. This study calls for implementing effective strategies to improve vaccination coverage at a national level while focusing on states with high levels of multidimensional poverty.

## Graphical abstract

Diphtheria vaccination coverage among Nigerian children remains suboptimal (56%). Coverage is significantly lower in Northern states and inversely correlated with multidimensional poverty. Addressing poverty‐related deprivations is essential to improving immunization equity and curbing recurrent diphtheria outbreaks.

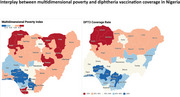

## 1 Introduction

Diphtheria constitutes a public health emergency of humongous scale in Nigeria, given its recurrent outbreaks. It is caused by Corynebacterium diphtheriae through the production of an exotoxin that is highly contagious and potentially fatal. The most recent diphtheria outbreak in Nigeria started in December 2022 when the Nigerian Center for Disease Control and Prevention (NCDC) was notified of a suspected diphtheria outbreak in Lagos and Kano states, the most populous states and economic centers based on World Health Organization [1]. As of October 3, 2023, there were 13,204 suspected cases and 8406 confirmed cases [2]. As of September 25, 2023, there were 453 recorded deaths, accounting for a case‐fatality rate of 6.3% [2]. About 73.7% of confirmed cases were among children aged 2–14 years, and 64% of confirmed cases occurred among individuals who were not fully vaccinated for diphtheria [3]. Similarly, the previous diphtheria outbreaks in 2011 had occurred in Borno state, and approximately 64% of its cases were among children aged <10 years, and 98% of cases were among individuals who have never been vaccinated against diphtheria [4].

Diphtheria vaccine is available through a DPT (diphtheria, pertussis, and tetanus) vaccine, which is part of the National Program on Immunization (NPI) in Nigeria. However, its vaccination coverage rate remains suboptimal, with a national coverage of 57% as per the Multiple Indicator Cluster Survey (MICS) that was conducted by the National Bureau of Statistics (NBS) and the United Nations International Children's Emergency Fund (UNICEF) in 2021 [1, 5]. Generally, diphtheria has a variable case‐fatality rate that can be as high as 40% in areas with high poverty levels [1]. According to the National Bureau of Statistics (NBS) data on multidimensional poverty, states, such as Kano, Katsina, Bauchi, Kaduna, Borno, and Yobe, where diphtheria outbreaks occurred, have scored high on multidimensional poverty index (MPI) [6]. MPI is an index that measures the proportion of households in a country deprived along three dimensions (monetary poverty, education, and basic infrastructure services), thereby depicting a more complete picture of poverty. Therefore, it reflects people's lived experiences beyond the dimension of income alone [7, 8].

Despite the availability of safe and effective diphtheria vaccines, Nigeria has experienced recurrent outbreaks in recent years, largely attributed to suboptimal immunization coverage and structural barriers to vaccine uptake. A recent comprehensive review of diphtheria in Nigeria highlights systemic, social, and environmental challenges that hinder vaccination efforts and facilitate outbreaks across multiple states [9]. Epidemiological analyses further indicate that declining DTP3 coverage, exacerbated by health system disruptions, including those related to the COVID‐19 pandemic, has increased vulnerability to diphtheria resurgence [10]. Sustainable improvements in routine immunization coverage are therefore critical for controlling diphtheria transmission and reducing disease burden in Nigeria.

To our knowledge, no attempt has been made to examine the diphtheria vaccination coverage in the context of multidimensional poverty in Nigeria. Prior studies have mainly described outbreaks [4, 11], assessed diphtheria vaccine coverage [12, 13], and factors associated with the vaccine uptake [14, 15]. Similarly, previous studies examined DPT coverage pre‐ COVID‐19 pandemic [12, 16]. The COVID‐19 pandemic substantially disrupted routine immunization services worldwide. WHO and UNICEF reported the largest sustained global decline in childhood vaccination coverage in approximately three decades, with DTP3 coverage falling between 2019 and 2021 [17]. These disruptions were attributed to lockdown measures, health system strain, diversion of resources, vaccine supply chain interruptions, and reduced healthcare utilization [18]. Nigeria was similarly affected, with increases in zero‐dose children and missed routine immunization appointments reported during the pandemic period [19]. Given the established relationship between suboptimal DTP3 coverage and diphtheria outbreaks, examining vaccination coverage during the COVID‐19 period provides important insight into population vulnerability to subsequent outbreaks. This research aims to (1) measure the crude and state‐specific DPT vaccination coverage during COVID‐19 pandemic in Nigeria (36 states and the Federal Capital Territory) and (2) examine the correlation between diphtheria vaccination coverage and multidimensional poverty score.

## 2 Methods

### 2.1 Study Population

Nigeria is a lower middle‐income country, Africa's largest economy, and most populous Black nation. With an estimated population of 225 million people, Nigeria is the sixth most populous country on the earth, with a median age of 17.2 years [20]. Nigeria has a predominantly young population, with approximately half of its population being less than 19 years old [21]. Levels of poverty and literacy vary among Nigerian states, but the Northern states generally have lower literacy and higher poverty levels [22]. Nigeria operates three levels of healthcare delivery, namely, primary, secondary, and tertiary. The primary and secondary levels of healthcare delivery are mainly responsible for vaccine administration; however, tertiary centers have joined to boost vaccine delivery among the population.

### 2.2 Design and Data Sources

This is a correlational (ecological) study that uses publicly available survey data from the 2021 MICS and National Immunization Coverage Survey (NICS) to examine the association between multidimensional poverty and diphtheria vaccination coverage at the state level. The present study did not involve direct sampling or data collection from individual participants but relied exclusively on aggregated state‐level estimates reported in the official survey publications.

Data on MPI were obtained from the National Bureau of Statistics (NBS), which computes MPI using multiple indicators reflecting deprivations in health, education, and living standards across the 36 states and the Federal Capital Territory.

Data on DPT vaccination coverage among children aged 12–23 months were obtained from the 2021 MICS/NICS report codeveloped by the NBS and UNICEF [5]. The vaccination coverage estimates were generated using the World Health Organization's Vaccination Coverage Quality Indicator (VCQI) software and represent weighted state‐level proportions of children who partially or fully completed DPT vaccination.

### 2.3 Inclusion and Exclusion Criteria

Children were included in the 2021 MICS/NICS if they were usual residents of selected households within sampled enumeration areas (EAs) across the 36 states and the Federal Capital Territory of Nigeria. For this study, the analytic sample was restricted to children aged 12–23 months at the time of the survey who had available information on DPT vaccination status, as documented by vaccination card or maternal/caregiver report.

EAs that were politically inaccessible due to insecurity were excluded from the NICS/MICS at the protocol stage.

### 2.4 Sampling

The sampling procedures described below pertain to the original 2021 MICS and National Immunization Coverage Survey (NICS), from which state‐level DPT vaccination coverage estimates were derived for this ecological analysis.

The 2021 MICS used a multi‐stage, stratified cluster sampling method to select the sample. The sampling frame was based on Nigeria's 2006 Population and Housing Census. The 36 states of Nigeria and the Federal Capital Territory were the sampling strata, making a total of 37 strata. Each stratum was divided into EAs that were further divided into households. At the first sampling stage, a systematic random sampling was used to select the EAs clusters from the list of EAs in each local government area (LGA). In the second sampling stage, a listing of households in each EA was made, and samples of households were selected, using probability proportional to size sampling.

The sample size for the 2021 MICS was calculated to be 1850 clusters (50 clusters in each state) with 37,000 households (1000 households from each state). To increase the number of children in the sample and improve the accuracy of the immunization indicators, an additional sample of 337 clusters with 6740 households was added from the National Immunization Coverage Survey. A total of 128 EAs (MICS = 95, NICS = 33) were politically inaccessible and therefore were not included. Overall, the final sample size was 201,942 participants; 29,409 of them were children under the age of 5 years, of which 5652 were in the age category 12–23 months. Hence, the final sample size for the current study was 5652 children.

### 2.5 Data Collection

Standard MICS questionnaires were administered, and data were collected using the computer‐assisted personal interviewing (CAPI) device. The interviewers asked the mothers or caretakers to show them the vaccination cards that the children received from health facilities. They recorded the vaccination information from the cards into the MICS questionnaire. If the child did not have a vaccination card, the interviewer asked the mother to remember if the child had received each of the vaccinations and how many doses for each one. The response rate among the households in the sample was 98.9% [5].

The survey protocol was approved by the Steering and Review Committees of the NBS in August 2021. The protocol outlined potential risks and strategies to mitigate these risks if they arise. In addition to verbal consent, written assents from children aged 15–17 years old and parental consents were obtained.

### 2.6 Study Measures

#### 2.6.1 Diphtheria Vaccination

The main outcome for this study is the diphtheria vaccination coverage. It is defined as the percentage of individuals 12–23 months old who were fully vaccinated (have received the DPT1, DPT2, and DPT3 doses) in each geographical state in Nigeria. As DPT3 is the last dose of the vaccines, DPT3 coverage rate in this study was used to indicate the proportion of children who completed the vaccines. Partial vaccination was defined as the percentage of children who received at least one dose of DPT (DPT1) but did not complete the three doses (missing DPT2 or DPT2 and DPT3) doses. This was calculated as the difference between the percentage who received DPT1 and the percentage who received DPT3. Unimmunized children were defined as those who did not receive any dose of DPT. Because this study used publicly reported, aggregated state‐level coverage estimates rather than raw individual‐level data, the percentage of unimmunized children was calculated as 100 minus the percentage of children who received at least one dose of DPT (DPT1). All coverage measures were expressed as percentages (0–100 scale).

#### 2.6.2 Multidimensional Poverty

The MPI is a composite measure developed by the United Nations Development Program (UNDP) and the Oxford Poverty and Human Development Initiative to assess deprivations across multiple dimensions of well‐being, including health, education, and living standards [23]. In Nigeria, the National Bureau of Statistics (NBS) computes state‐level MPI estimates using fifteen indicators reflecting deprivations, such as nutrition, school attendance, years of schooling, housing conditions, access to water and sanitation, cooking fuel, employment, and security shocks.

For this study, we used the state‐level MPI estimates published by the NBS in 2022. These values represent aggregated proportions of individuals classified as multidimensionally poor in each state and the Federal Capital Territory. The MPI values were derived by the NBS using the standard formulation:

MPI=H×A

where *H* represents the incidence (proportion of the population identified as multidimensionally poor), and *A* represents the intensity (average proportion of deprivations experienced by poor individuals).

The present study did not perform any new MPI calculations but relied exclusively on the published state‐level MPI estimates reported by the NBS.

### 2.7 Data Analysis

Categorical variables were summarized using frequency measures, and continuous variables were summarized using mean and standard deviation. The overall average vaccination coverage in Nigeria was calculated as the weighted average vaccination coverage in all states. Correlation analysis was used to examine the relationship between states DPT full vaccination coverage rates and their corresponding MPI scores. All statistical analyses were done using SPSS version 28. Alpha level was set to 0.05.

The 2021 MICS sample is not self‐weighted, as different sampling fractions were applied in each state. To ensure accurate representation, weights were calculated on the basis of sampling fraction, nonresponse rates, and adjustment factor for each state (stratum). Details about weight calculations can be found elsewhere [5].

## 3 Results

**FIGURE 1:**
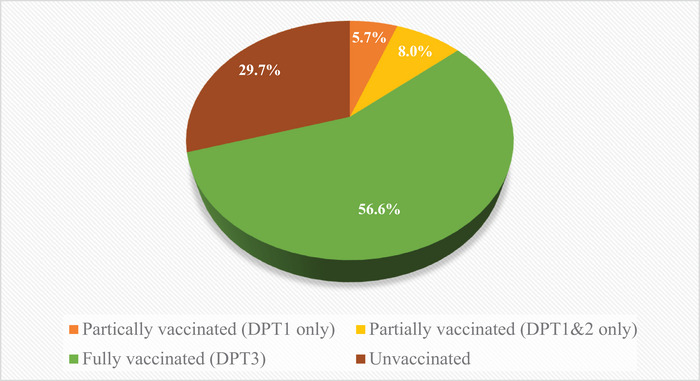
DPT vaccination coverage status among children (12–23 months) in Nigeria from 2021 Multiple Indicator Cluster Survey and National Immunization Coverage Survey. DPT, diphtheria–pertussis–tetanus.

The survey included 5652 children between 12 and 23 months old, of which 2861 (50.6%) were males and 2791 (49.3%) were females. In overall, the percentage of children who received at least one dose of DPT vaccine was 70.3%, of which 80.5% completed the three vaccine doses, 11.4% completed two doses, and 8.1% completed only one dose. In overall, 56% of the surveyed children have completed the three doses of DPT vaccine (fully vaccinated). The percentage of children who received partial DPT vaccination by taking only the first or first and second dose was 13.7% (details in Figure 1).

**FIGURE 2:**
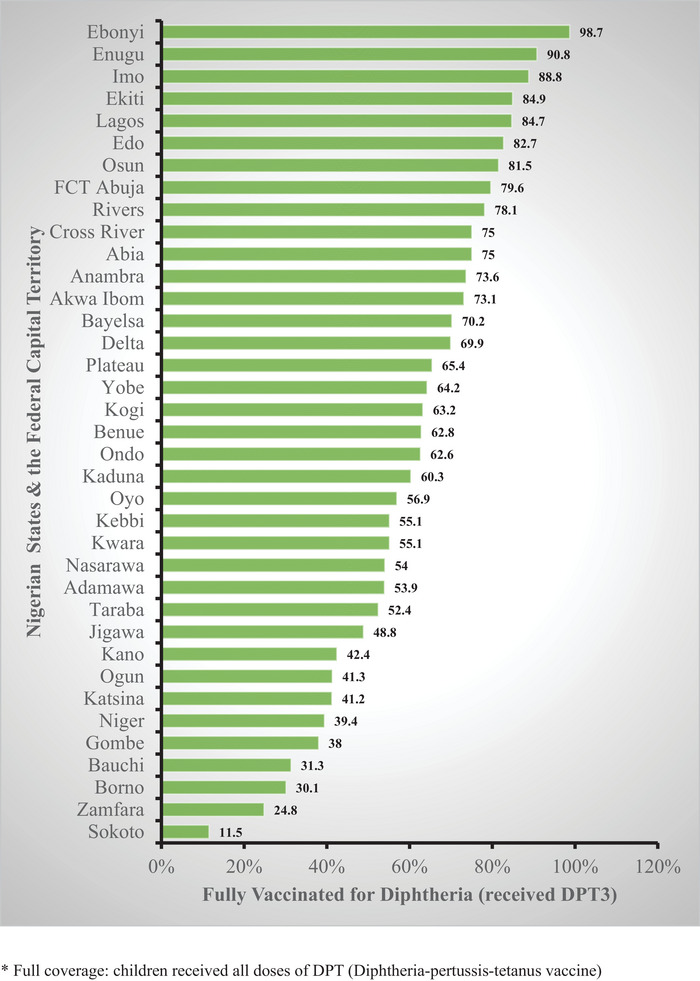
Full diphtheria vaccination coverage rate (DPT3). Across Nigerian states among ages 12–23 months, from 2021 Multiple Indicator Cluster Survey/National Immunization Coverage Survey. Full coverage: children received all doses of DPT (diphtheria–pertussis–tetanus vaccine). DPT, diphtheria–pertussis–tetanus.

As Figure 2 shows, DPT full vaccination coverage rate in 2021 varies across Nigerian states. The seven states that had 80% full DPT coverage rate or higher were Ebonyi, Enugu, Imo, Ekiti, Lagos, Edo, and Osun, whereas those with less than 50% full coverage were Sokoto, Zamfara, Borno, Bauchi, Gombe, Niger, and Katsina. The Federal Capital Territory has a DPT full coverage of 79.6%. Ebonyi state has the highest DPT full vaccination coverage (98.7%), whereas Sokoto state has the lowest (11.5%).

**FIGURE 3:**
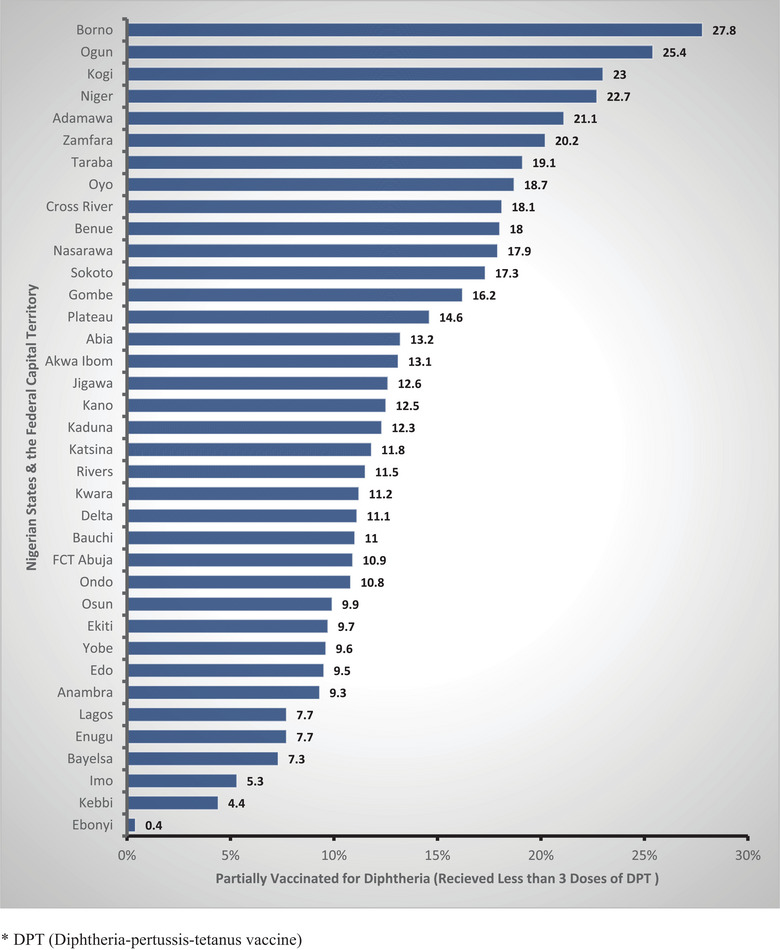
DPT partial vaccination rates across Nigerian states among ages 12–23 months, from 2021 Multiple Indicator Cluster Survey/National Immunization Coverage Survey. DPT, diphtheria–pertussis–tetanus vaccine.

Findings regarding un‐vaccination showed that Ebonyi (0.9%) and Enugu (1.5%) had the lowest un‐vaccination rates, whereas Sokoto had the highest (71.2%). Similarly, Ebonyi had the lowest partial (i.e., received less than three doses of DPT) vaccination rates (0.4%), and Borno had the highest (27.8%). Borno, Ogun, Kogi, Niger, and Adamawa states had the highest partial DPT vaccination rates, whereas Ebonyi, Kebbi, Imo, Bayelsa, and Enugu had the lowest (Figure 3).

**FIGURE 4:**
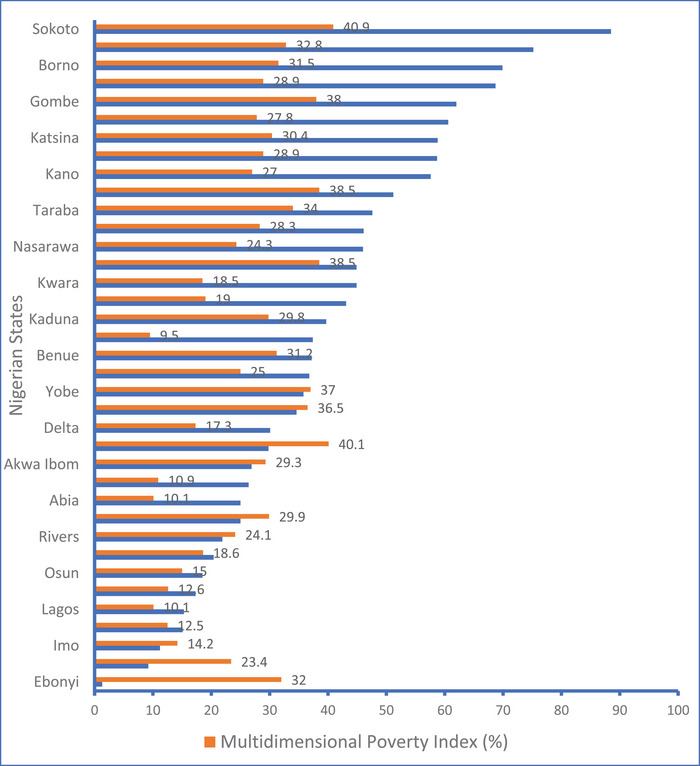
The percentage of children 12–23 months who did not receive or partially received the DPT vaccine and the multidimensional poverty index across the Nigerian states in 2021 from Multiple Indicator Cluster Survey and National Immunization Coverage Survey.

**FIGURE 5:**
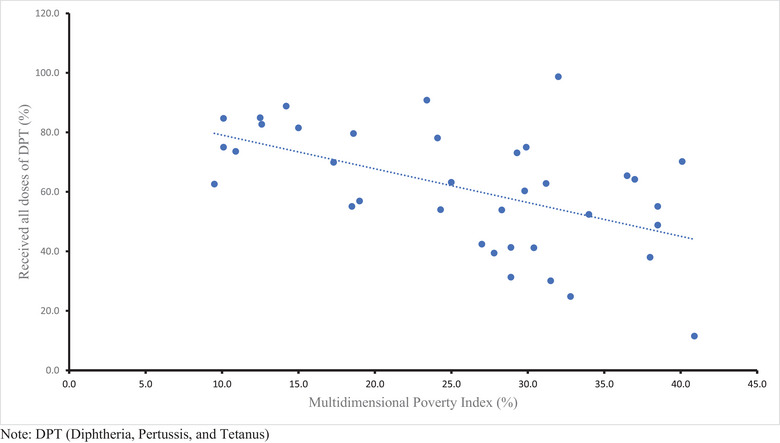
Correlation between DPT3 vaccination rate and multidimensional poverty index among children 12–23 months across Nigerian states from Multiple Indicator Cluster Survey and National Immunization Coverage Survey (2021). DPT, diphtheria, pertussis, and tetanus.

As Figure 4 shows, MPI was the highest in Sokoto, Bayelsa, Jigawa, Kebbi, and Gombe, and the lowest was in Ondo, Lagos, Abia, Anambra, and Ekiti states. These states with lower MPI had lower proportion of unvaccinated and partially vaccinated children. However, Ebonyi state, despite having a high MPI, had the lowest rate of unvaccinated and partially vaccinated children. Figure 5 shows the correlation between DPT full coverage and MPI. There was a statistically significant moderate negative correlation between DPT full vaccination coverage and MPI (*r* = −0.534, *p* = <0.001).

## 4 Discussion

This study reveals substantial state‐level disparities in DPT3 vaccination coverage across Nigeria and demonstrates a significant inverse association between multidimensional poverty and immunization performance. The findings highlight persistent regional inequalities, with northern states disproportionately affected by lower coverage levels. Beyond documenting suboptimal vaccination rates, the study underscores the structural role of multidimensional deprivation in shaping immunization outcomes during the COVID‐19 period. These results suggest that improving vaccination coverage in Nigeria requires interventions that address broader social and systemic determinants rather than focusing solely on individual‐level behaviors.

The 2021 survey period coincided with the global COVID‐19 pandemic, which substantially disrupted routine immunization services worldwide. WHO and UNICEF reported a decline in global DTP3 coverage between 2019 and 2021, marking the largest sustained backslide in childhood vaccination in several decades [17]. In Nigeria, pandemic‐related disruptions—including lockdown measures, movement restrictions, diversion of health resources toward COVID‐19 response, fear of infection in health facilities, and temporary suspension of outreach immunization services—likely compounded preexisting structural barriers to vaccine access [18, 24].

Although immunization coverage in Nigeria had been suboptimal prior to the pandemic, the 2021 coverage estimate of 56% remains lower than global targets and suggests persistent vulnerability during the pandemic recovery phase. Reports during the pandemic period also indicated increases in zero‐dose children and missed immunization appointments, particularly in northern Nigeria [19]. Therefore, the findings of this study reflect not only longstanding socioeconomic and geographic disparities but also the potential amplification of these inequities during the COVID‐19 period.

Importantly, several barriers to vaccination in Nigeria predate the COVID‐19 pandemic, including insecurity in northern states, limited health infrastructure, poverty‐related access challenges, and vaccine hesitancy [25, 26, 27]. However, the pandemic may have exacerbated these barriers by restricting mobility, disrupting supply chains, and intensifying misinformation surrounding vaccines. Pre‐pandemic national DPT3 coverage estimates in Nigeria ranged between approximately 50% and 57% in recent survey cycles, indicating that coverage was already below optimal levels [1, 5]. However, global evidence suggests that COVID‐19 further stalled progress and increased the number of zero‐dose children [19]. Thus, although low coverage is not new in Nigeria, the pandemic period may have impeded recovery efforts and deepened disparities across states.

This study finding of suboptimal DPT vaccination coverage among Nigerian children was consistent with previous studies that revealed low vaccination coverage among children in Nigeria [25, 28, 29]. The factors previously identified as possible reasons for the low vaccination coverage in Nigeria were vaccine safety concerns, low maternal education, and inadequate information about vaccines [28]. Over time, there have been concerns and misconceptions about vaccine safety, as some caregivers have attributed vaccine to the development of rickets in children and reduced reproductive potential when children become older [30, 31]. The fear that vaccines may result into infertility in adulthood is popular myth that scares the guardians from the vaccines. Previous research indicated poor knowledge regarding vaccine‐preventable diseases among the Nigerian mothers from Enugu state [32]. Poor knowledge may also explain the partial vaccination coverage in the states with high partial vaccination coverage, as people may not be aware of the importance of completing the vaccine doses.

This study showed variations in DPT vaccination coverage by states. Higher vaccination coverage was observed in Southern than Northern Nigeria's states. These north–south vaccination coverage disparities in Nigeria were previously reported for other national vaccines, including Bacillus Calmette‐Guerin, measles vaccines, and oral polio vaccine [24, 33]. These variations were previously attributed to socioeconomic status, lower levels of educational attainment, deprivations in healthcare access, higher levels of insecurity, and lower vaccine literacy [24, 26, 33, 34, 35, 36].

Additionally, the interrupted healthcare access due to the political insecurity and conflicts in the Northern part of Nigeria [37, 38] may interpret the difference in vaccination coverage between the two regions. On the basis of anecdotal evidence, people who live in the Northern states, such as Moslems and Fulani, had low vaccination coverage rates [30, 33, 39]. This may in part interpret the recurrent outbreak of vaccine‐preventable diseases in those areas [26].

An important finding of this study was the exceptional DPT3 vaccination coverage observed in Ebonyi state despite its relatively high MPI. This suggests that poverty alone does not fully determine immunization performance at the state level [25, 40]. Several contextual factors may explain this divergence. Southeastern states, including Ebonyi, have historically demonstrated stronger routine immunization performance, potentially reflecting more stable security conditions, higher maternal literacy rates, greater vaccine acceptance, and stronger primary healthcare engagement compared to some northern states [16, 24, 33]. In addition, local governance quality, effective outreach programs, and community‐level mobilization may mitigate the impact of economic deprivation on vaccination uptake [27, 34, 41]. Similar north–south disparities in immunization coverage have been reported in previous national surveys, indicating that sociocultural and structural determinants beyond poverty influence vaccine coverage patterns in Nigeria [25, 33, 40].

Although previous research had investigated the vaccination coverage in the context of socioeconomic factors [27, 40, 42, 43], this study was unique in using the MPI as a sustainable development construct that measures various deprivations that people experience and accounts for all those factors. Poor households, for instance, had higher odds of being unvaccinated or receiving partial vaccination [42]. Similarly, children of parents with higher educational level and income had higher vaccination coverage than those for lower education [29, 41, 44, 45]. Additionally, accessibility to and affordability of healthcare are more likely to be lower among people with lower socioeconomic class [29], and this may contribute to low vaccination coverage in general [25].

The findings of this study have implications on immunization program planning and implementation in Nigeria. Interventions to scale up vaccination coverage must be multidimensional, accounting for the deprivations that Nigerians encounter in terms of access to health, education, and living standards. There should be an improvement in vaccine education among caregivers as many of them were found to have insufficient knowledge of the importance of vaccination [43]. Additionally, inaccessible roads and long travel times are among the strong factors that may disrupt the vaccination clinics attendance [27]. Improving road networks, widening vaccination networks, and using mobile vaccination services may improve access to vaccination and increase coverage in Nigeria. As religion has been highlighted as a strong factor for vaccination, it might be helpful if the Nigerian government partners with religious organizations to assist in raising the level of awareness and facilitate immunization delivery to those populations.

This study provided clear insight about the vaccination coverage of diphtheria through the DPT vaccine, which provides an additional information about pertussis and tetanus vaccines. In addition, this study is the first to explore the correlation between MPI as a construct of sustainable development and DPT vaccination coverage in Nigeria. However, this study has few limitations. First, underrepresentation due to the political inaccessibility of 27 LGAs in Borno state, which is equivalent to nearly 70% of the state's population based on a 2018 census. Though this did not account for people who moved from insecure to secure LGAs, and the analysis was weighted to account for underrepresentation and missing information. Second, the estimates of vaccination coverage are based on both the card and the mother's report of the child's vaccinations. The latter is subject to recall bias. Nonetheless, maternal reporting of vaccination has previously shown to be a valid reporting approach with low recall bias [46]. Finally, although there was a linear correlation between vaccination coverage and MPI at states’ levels, this does not necessarily imply a correlation at individual level. There is no information in this study to support that children for families with higher MPI had lower vaccination coverage than from those with lower MPI for instance. Hence, the correlation between MPI and vaccination coverage is subject to an ecologic fallacy [47].

## 5 Conclusions

The overall vaccination coverage in Nigeria was suboptimal. Northern states had lower diphtheria vaccination coverage than southern states. There is a negative correlation between MPI and DPT full vaccination coverage. To our knowledge, this is one of the first ecological studies in Nigeria to explicitly examine the relationship between multidimensional poverty and DPT vaccination coverage using nationally representative data. Findings from this study may inform national immunization agencies and nonprofit organizations to adopt a multidimensional strategy to improve vaccination coverage in states with high multidimensional poverty, considering the diverse deprivations that populations experience across health, education, and living conditions.

## Author Contributions

**Olufemi Olulaja**: conceptualization, methodology, data analysis, writing (original draft), visualization. **Rana Jaber**: provided extensive leadership and guidance for the study, critical supervision for data analysis, and substantial review and interpretation for the study findings. She critically revised the manuscript and approved the final draft for submission.

## Funding

The authors have nothing to report.

## Ethics Statement

Ethical approval was sought from the Office of Research Compliance, Integrity, and Safety at Northern Illinois University, DeKalb, Illinois, which adjudged that this study does not meet the definition of human subject research and is not subject to oversight by the Institutional Review Board (Protocol Number: HS24‐0175). Data used in this ecological study is publicly available.

## Consent

We, the authors of this manuscript, hereby confirm that we have read and approved the final version of this article titled “Diphtheria Vaccination Coverage and Correlation with Multidimensional Poverty among Children in Nigeria: A Correlational Study from Multiple Indicator Cluster Survey and National Immunization Coverage Survey 2021”. We also consent to its submission and publication in Public Health Challenges.

## Conflicts of Interest

The authors declare no conflicts of interest.

## Data Availability

Data used in this ecological study are provided by National Bureau of Statistics and the United Nations Children's Fund and are publicly available at. https://www.unicef.org/nigeria/media/6316/file/2021%20MICS%20full%20report%20.pdf and https://www.nigeriapovertymap.com/explorempi, respectively.
